# Development of Cerebellar Reserve

**DOI:** 10.3390/cells11193013

**Published:** 2022-09-27

**Authors:** Hiroshi Mitoma, Shinji Kakei, Mario Manto

**Affiliations:** 1 Department of Medical Education, Tokyo Medical University, Tokyo 160-0023, Japan; 2 Department of Anatomy and Physiology, Jissen Women’s University, Tokyo 191-8510, Japan; 3 Service de Neurologie, Médiathèque Jean Jacquy, CHU-Charleroi, 6000 Charleroi, Belgium; 4 Service des Neurosciences, University of Mons, 7000 Mons, Belgium

**Keywords:** cerebellum, cerebellar reserve, cerebellar development

## Abstract

The cerebellar reserve is defined as the capacity of the cerebellum for compensation and restoration following injury. This unique cerebellar ability is attributed to various forms of synaptic plasticity that incorporate multimodal and redundant cerebellar inputs, two major features of the cerebellar circuitry. It is assumed that the cerebellar reserve is acquired from the age of 12 years after the maturation of both the cerebellar adaptative behaviors and cerebellar functional connectivity. However, acquiring the cerebellar reserve is also affected by two other factors: vulnerability and growth potential in the developing cerebellum. First, cerebellar injury during the critical period of neural circuit formation (especially during fetal and neonatal life and infancy) leads to persistent dysfunction of the cerebellum and its targets, resulting in the limitation of the cerebellar reserve. Secondly, growth potential appears to facilitate cerebellar reserve during the stage when the cerebellar reserve is still immature. Based on these findings, the present mini-review proposes a possible developmental trajectory underlying the acquisition of cerebellar reserve. We highlight the importance of studies dedicated to the understanding of the cerebellar resilience to injuries.

## 1. Introduction

The cerebellar reserve is defined as the capacity of the cerebellum for compensation and restoration following pathological changes affecting this part of the brain [1,2,3,4]. Cerebellar reserve constitutes remarkable inherent forces of resilience to pathologies and ability for recovery, taking advantage of the huge number of neurons and glial cells present in the cerebellum. This description dates back to the classic paper by Sir G. Holmes [5]. Notably, cerebellar reserve and cerebellar learning share common mechanisms, including multiple forms of synaptic plasticity and multimodal afferents from the periphery and the cerebral cortex [3].

To the best of our knowledge, there is little or no information available on the development of the cerebellar reserve, despite the importance of cerebellar reserve in the physiology of sensorimotor functions. The aim of this mini-review is to provide details on the developmental process of the cerebellar reserve. After providing a summary of the clinical and physiological features of the cerebellar reserve, we first review the age-dependent developmental stages of the clinical setting of ataxia rating scales and adaptive locomotor behavior. Then, we highlight the developmental trajectory of cerebellar functional connectivity and synaptic plasticity. The final section is devoted to the construction of our concept of the development of the cerebellar reserve.

We searched papers related to “development of cerebellar reserve” from three perspectives. (1) We performed the search in PubMed using the key words: “cerebellum” AND “development of motor adaptation” AND “children”. After examining the abstracts in these 37 articles, we found literature of [6]. (2) We performed the search in PubMed using the key words: “Age-related reference” AND “ataxia scale”. After examining the abstracts of these 20 articles, we found [7]. (3) We finally performed the search in PubMed using the key words: “Physical therapy” AND “ataxia” AND “children” AND “systematic review”. Based on the contents of these papers, we proposed the development of cerebellar reserve and matched this with the basics of cerebellar development. To summarize the development of cerebellar circuits, we used review articles published in 2019–2022 [8,9].

## 2. Clinical Features and Physiology of Cerebellar Reserve

### 2.1. Types of Cerebellar Reserve in a Clinical Setting

There are two types of cerebellar reserve, depending on the etiology of the insult. First, in the case of restricted and transient lesions (e.g., stroke or traumatic damage), the unaffected cerebellar area compensates for lost functions (structural cerebellar reserve) [2]. On the other hand, in the case of extensive and progressive lesions (e.g., metabolic or degenerative pathology), the affected cerebellar area itself replenishes disorganized functions (functional cerebellar reserve) [2].

### 2.2. Underlying Physiology of Cerebellar Reserve

The concept of the *reserve* has also been proposed in other neurodegenerative diseases, such as cognitive reserve in Alzheimer’s disease [10,11], and motor reserve in Parkinson’s disease [12]. Compared with these types of reserves, the cerebellar reserve shows outstanding capacities for compensation and restoration following injury. Such a wealth of capacity appears to depend on the cerebellum-specific structural and functional features that are most probably unavailable in other regions of the central nervous system (CNS).

Among the 85–100 billion neurons of the whole brain, no fewer than 60% are located in the cerebellum, which comprises only about 10% of the brain mass [13,14]. The vast cerebellar neuronal networks are organized to function as an internal forward model that integrates an efference copy and afferent inputs to predict the future state of the motor apparatus to optimize motor control [15,16,17,18]. It is likely that the cerebellar reserve is based on two distinctive mechanisms inherent in the cerebellar circuits: (A) “redundant afferents to microzone” and (B) “multiple forms of synaptic plasticity”, as will be explained below.

#### 2.2.1. Redundant Afferents to Microzone

The cerebellum integrates two distinct lines of inputs required for an internal forward model, which are conveyed through mossy fibers (MFs): (1) an efferent copy from the controller; (2) a sensory feedback signal that encodes the past (~100 ms) state of the motor plant [17]. The functional unit of the cerebellar cortex is hypothesized as a microzone, a thin rostrocaudal strip of the cerebellar cortex [19]. Purkinje cells (PCs) in the same microzone receive climbing fiber (CF) input from a small group of neurons within a limited area in the inferior olivary nucleus. In turn, these PCs project to a small group of neurons in a deep cerebellar nucleus. It should be noted that the branching patterns of individual MFs are widely divergent along the mediolateral axis [20]. In addition, mediolateral orientation of the parallel fibers (i.e., granule cell (GC) axons) further increases the mediolateral extension of MF inputs. Individual GCs receive convergent multimodal (somatosensory, auditory, and visual) inputs via distinct MFs [21]. Therefore, MF inputs are highly convergent and divergent to individual microzones [3]. On the other hand, there is a basic arrangement of cortico-nuclear projections. Namely, PCs in the vermis project to the medial nucleus, PCs in the intermediate zones project to the interpositus nucleus, and PCs in the lateral hemispheres project to the dentate nucleus [22].

#### 2.2.2. Multiple Forms of Synaptic Plasticity

The multiple forms of plasticity in the cerebellar cortex appear to cooperate synergetically, generating the optimal cerebellar [23]. For example, rebound potentiation (RP) at GABAergic synapses on PC provides learning with more coarse spatial resolution than that of parallel fiber (PF)-PC long-term depression (LTD) [3]. Taken together, it is likely that spatially coarse learning by RP may be beneficial for succeeding spatially finer learning with LTD [3].

In summary, multimodal cerebral and peripheral inputs are integrated through multiple plastic changes at widely distributed synapses in the cerebellar cortex, which leads to optimization of the internal model. While these unique characteristics underlie cerebellar learning, at the same time, they give the cerebellum a surprising resistance to deficits.

## 3. Development of Cerebellar Contributions in Predictive Motor Control and Adaptive Locomotor Behavior

There are several arguments for a developmental trajectory of the cerebellar reserve, highlighting that the cerebellar reserve is a dynamic process maturing through the years of life.

### 3.1. Age-Dependent Changes in Clinical Rating Scales

The scores of various ataxia rating scales, such as the International Cooperative Ataxia Rating Scale (ICARS) and the Scale for Assessment and Rating of Ataxia (SARA), are significantly higher in children up to the age of 12 years, compared with the adults [7,24]. The high score suggests underdevelopment and immaturity of the cerebellar predictive online control during childhood [17,18].

### 3.2. Age-Dependent Development of Adaptive Locomotor Behavior

The developmental trajectory associated with cerebellar learning has been well investigated in studies on locomotor behavior. Infants gradually acquire sufficient strength and balance for locomotion and finally gain the ability to adapt their locomotor decisions to variations in the environment and changes in their bodily propensities [25]. Sathyanesan and Gallo defined adaptive locomotor behavior as associative conditioned learning involving locomotor adaptation in response to a goal-disruptive perturbation [26]. They categorized two features of the adaptive locomotor behavior: lower-level and higher-level features. The former includes coordination of posture and stepping, whereas the latter includes task-based learning in which movement coordination is necessary to adapt elemental movements to external conditions and limitations [26]. For example, task-based learning is observed when an infant learns how to climb stairs, in which infants need to coordinate sequential stepping and trunk movements to climb stairs. Another example is when an adolescent learns to dribble a soccer ball, avoiding opponents who get in his way. The cerebellum plays a critical role in these adaptive locomotor behaviors as explained below:

#### 3.2.1. Cerebellar Contribution to Lower-Level Adaptation

Children with genetic cerebellar ataxia, such as Friedreich’s ataxia and ataxia telangiectasia, display marked impairment of gait. Such impairment is due to several mechanisms, including deficits in multisensory integration in the cerebellum [27]. Thus, injury of the developing cerebellum highlights the importance of cerebellar circuitry to lower-level features in adaptive locomotor behavior. 

#### 3.2.2. Cerebellar Contribution to Higher-Level Adaptation

The split-belt treadmill walking task was used to examine the adaptation of stepping patterns to environmental changes [28]. The split-belt treadmill allows movement of each leg at different speeds at the time of immediate reaction, such that the slower leg spends more time in stance while the faster leg spends less time. Switching to normal conditions reverses the adaptative stepping to a normal one. Clinical studies have shown that during the early phase of the post-split-belt condition, the relative stance times were more asymmetrical in the cerebellar patient group (patients with stable focal lesions after cerebellar tumor resection) than in the control group [29]. Cerebellar patients were capable of adjusting locomotion in a feedback control manner, similar to the healthy subjects, but they failed to adapt their movements to predictive changes, highlighting the critical role of the cerebellum in this task [30].

#### 3.2.3. Age-Dependent Development of Adaptive Locomotor Behavior

Sathyanesan and Gallo indicated that lower-level features develop from birth to 10 years, while the higher-level features develop from early childhood to adolescence [26]. Another study showed that children aged 11 years consistently failed to adapt to the split-belt treadmill paradigm compared with the adults [6].

In conclusion, various cerebellar functions, including predictive motor control and adaptive locomotor behaviors, do not seem to mature until the age of 10 or 12.

## 4. Development of the Cerebellar Circuits

Neurobiological studies focusing on development have highlighted the dynamics of cellular proliferation and organization.

### 4.1. Cerebellar Neuronal Proliferation, Migration, and Myelination

During fetal development, the PCs first appear at embryonic (E) day 13–16 in mice and gestational week 7–13 in humans, which is followed by the formation of the PC plate [8]. PCs show extensive complexity in both dendritic length and arborization at P0 in mice and gestational week 28 in humans [8]. On the other hand, GCs initially proliferate in the external granular layer (EGL) at E17.5 up to P16 in mice, followed by migration of these cells into the internal granular layer (IGL) until P20 [8]. In humans, the formation of the EGL begins at gestational week 10 [8]. Then, at postnatal months 12–24, the EGL gradually disappears following decreased proliferation and migration of granule neurons into the IGL [9].

As the cerebellar cortex develops, the deep cerebellar nucleus also projects to target regions. The cerebellar nucleus neurons are identified at gestational week 16 in humans [31]. The superior cerebellar peduncle, which contains fibers from the dentate nucleus to the contralateral red nucleus and thalamus, develops in humans between the 28th week and the 6th month [9].

The human cerebellum undergoes the highest rate of growth during the first three months of life (more than doubling in size) [32], due to a combination of synaptogenesis (enlargement and increased arborization of PC dendrites) and birth and migration of new granular neurons, thereby enlarging both the molecular and granular layers of the cortex. Furthermore, white matter of the cerebellum is among the first regions of the brain to myelinate [9]. The myelination is high during the third trimester and continues between the ages of 2 and 5 years [33].

### 4.2. Development of Synapse Formation and Functional Connectivity with Other Regions

The first two postnatal weeks in mice and the first 12 months of life in humans are critical for synapse formation in the cerebellum [8,34], including pruning (e.g., CF-PC synapses) [35], remodeling of CF terminals (e.g., changes from an immature configuration with a dense aggregation of swellings to a mature configuration) [36], and PC axons targeting cerebellar nuclei [37]. Experimental studies involving targeted deletion of the mGluR1 gene demonstrated abnormal regression of multiple CF innervation, impaired LTD, and abnormal motor coordination [38]. Thus, it seems that synaptic pruning and synaptic plasticity share common molecular machinery [39,40], suggesting the simultaneous maturation of CF innervation and CF plasticity.

In addition to the above investigations, structural imaging studies indicated a steady increase in cerebellar size after birth, which continues until late childhood to adolescence [41,42]. Interestingly, the total volume of the cerebellum followed an inverted U-shaped developmental trajectory, reaching a peak level at age 11.8 years in females and 15.6 years in males [42]. The peak of the developmental trajectory of the cerebellum was noted to occur earlier in the inferior posterior lobe and later in the superior posterior and anterior lobe, although significant differences were not observed [41]. In contrast, the vermis volume remained the same during the same age period [41]. This regional specificity suggests that functional connectivity to extracerebellar regions might influence cerebellar morphological development. Consistent with this notion, a solid functional connectivity corresponding to the sensory-motor system was observed in infants, whereas associations with executive control and default mode systems also developed in children and adults [43].

In conclusion, the cerebellum shows steady postnatal development. The developmental trajectory appears to differ in synaptic plasticity and functional connectivity compared to other CNS regions. Synaptic plasticity matures during the first 12 months of life. In contrast, the exact completion time of functional connectivity seems to depend on the cerebellar region, spanning from infancy to late childhood. Therefore, immature functional connectivity might be one reason for the clumsiness in cerebellum-mediated adaptive locomotor behaviors during childhood.

## 5. Development of Cerebellar Reserve

### 5.1. Determinants of the Cerebellar Reserve during Development

In adults, the cerebellar reserve is attributed to integrating multimodal cerebral and peripheral inputs through multiple forms of synaptic plasticity (Section 1). Thus, it is assumed that cerebellar reserve is definitely acquired following maturation of the cerebellum-mediated adaptative behaviors and completion of the cerebellar functional connectivity (at least beyond 12 years of age). Consistently, imaging studies suggest that the functional connectivity between the cerebellum and cerebral cortex develops during late childhood to adolescence. Interestingly, one systematic review confirmed the effectiveness of physical rehabilitation on CAs in adolescence [44].

However, the real situation appears to be more complex. Cerebellar maturation is protracted over a broad time window after birth [8] (Section 2 and Section 3). In addition to this developmental feature, the cerebellar reserve is also influenced in the developing cerebellum by two additional factors: vulnerability and growth potential. The balance between vulnerability and growth potential determines cerebellar reserve in the developing cerebellum.

#### 5.1.1. Vulnerability in the Absence of Cerebellar Reserve

Sathyanesan et al. proposed that the human cerebellum has a protracted developmental timeline, thus broadening the window of vulnerability to neurological disorders [8]. Importantly, cerebellar damage during the critical period of circuit formation (especially fetal and neonatal life and infancy) could lead to persistent dysfunction of the cerebellum and its target areas, such as the spinal cord, brainstem, and cerebral cortex [45]. During this vulnerable period, the cerebellum has no reserve for overcoming the pathological changes.

#### 5.1.2. Growth Potential Facilitating Reserve

The therapeutic benefits of rehabilitation have been reported in early childhood, at the age when adaptive locomotor behaviors have not yet matured [6]. These studies suggest that in the absence of full cerebellar reserve development, the cerebellar growth potential can facilitate the acquisition of reserve capacity.

### 5.2. Differences in the Maturity of Cerebellar Reserve between Sensory-Motor and Cognitive Domains

The functional connection between the cerebellum and the sensorimotor cortex develops in infants, but the functional connection with the cognitive cortices develops during childhood and adulthood (Section 3), suggesting that the time of acquisition of the cerebellar reserve capacity occurs later in the cognitive domain compared with the motor-sensory domain. Consistent with this expansion of the vulnerable stage in the cognitive domain, it is well known that children with impaired cerebellar growth (e.g., neonates with brain injury and children who undergo surgical excision of tumor) can show conspicuous non-motor deficits, such as impaired executive function, memory, and language, without conspicuous motor symptoms [8]. For example, lesions in the hemisphere elicited the language-delay and the deficits in visual and verbal reasoning, whereas damages to the vermis caused withdrawn social behavior, impaired gaze, anxiety, and stereotyped behaviors [46]. Thus, the cerebellar reserve should be evaluated separately in the sensory-motor and cognitive domains.

## 6. Conclusions

The cerebellum undergoes postnatal development of intra-cerebellar circuits and synaptic functions, as well as functional connectivity with other CNS regions. In the developing cerebellum, the acquisition of cerebellar reserve can be influenced by three factors, (1) vulnerability, (2) growth potential, and (3) completion of integrating multimodal inputs through various forms of synaptic plasticity. Physiological and clinical studies are needed to confirm the above scenarios. This hypothesis is derived from very few fragmentary data, including the age-dependent development of adaptive locomotor behavior, the imaging studies on functional connectivity, and the few rehabilitation data.

Thus, useful data might also be obtained from functional MRI studies that trace changes in cerebellar re-organization following cerebellar injury in children. Given the numerous forms of CAs encountered in the clinic and their distinct functional impacts from the early phases of life to the elderly, it is anticipated that the re-organization of the cerebellar reserve evolves dynamically through the various stages of development and in a different manner according to the location of the lesion. Studies should be performed to elucidate how CAs impact on the repertoire of resilience inherent to the cerebellar circuitry. Cerebellar research has highlighted the importance of cerebellar reserve [47] but the concept has likely been overlooked.

## Data Availability

The concept reported in this manuscript is not associated with raw data.

## References

[1] Mitoma H., Manto M., Hampe C.S. (2018). Time is cerebellum. Cerebellum.

[2] Mitoma H., Buffo A., Gelfo F., Guell X., Fucà E., Kakei S., Lee J., Manto M., Petrosini L., Shaikh A.G. (2019). Consensus paper. Cerebellar reserve: From cerebellar physiology to cerebellar disorders. Cerebellum.

[3] Mitoma H., Kakei S., Yamaguchi K., Manto M. (2021). Physiology of cerebellar reserve: Redundancy and plasticity of a modular machine. Int. J. Mol. Sci..

[4] Manto M., Kakei S., Mitoma H. (2021). The critical need to develop tools assessing cerebellar reserve for the delivery and assessment of non-invasive cerebellar stimulation. Cerebellum Ataxias.

[5] Holmes G. (1917). The symptoms of acute cerebellar injuries due to gunshot injuries. Brain.

[6] Vasudevan E.V., Torres-Oviedo G., Morton S.M., Yang J.F., Bastian A.J. (2011). Younger is not always better: Development of locomotor adaptation from childhood to adulthood. J. Neurosci..

[7] Lawerman T.F., Brandsma R., Burger H., Burgerhof J.G.M., Sival D.A., the Childhood Ataxia and Cerebellar Group of the European Pediatric Neurology Society (2017). Age related reference values for the pediatric Scale for Assessment and Rating of Ataxia: A multicentre study. Dev. Med. Child Neurol..

[8] Sathyanesan A., Zhou J., Scafidi J., Heck D.H., Sillitoe R.V., Gallo V. (2019). Emerging connections between cerebellar development, behavior, and complex brain disorders. Nat. Rev. Neurosci..

[9] De Benedictis A., Rossi-Espagnet M.C., de Palma L., Carai A., Marras C.E. (2022). Networking of the human cerebellum: From anatomo-functional development to neurosurgical implications. Front. Neurol..

[10] Stern Y. (2012). Cognitive reserve in aging and Alzheimer’s disease. Lancet Neurol..

[11] Stern Y. (2017). An approach to studying the neural correlates of reserve. Brain Imaging Behav..

[12] Palmer S.J., Ng B., Abugharbieh R., Eigenraam L., McKeown M.J. (2009). Motor reserve and novel area recruitment: Amplitude and spatial characteristics of compensation in Parkinson’s disease. Eur. J. Neurosci..

[13] Colin F., Ris L., Godaux E., Manto M., Pandolfo M. (2002). Neuroanatomy of the cerebellum. The cerebellum and Its Disorders.

[14] Walloe S., Pakkenberg B., Fabricius K. (2014). Stereological estimation of total cell numbers in the human cerebral and cerebellar cortex. Front. Hum. Neurosci..

[15] Wolpert D.M., Ghahramani Z., Jordan M.I. (1995). An internal model for sensorimotor integration. Science.

[16] Popa L.S., Hewitt A.L., Ebner T.J. (2013). Purkinje cell simple spike discharge encodes error signals consistent with a forward internal model. Cerebellum.

[17] Tanaka H., Ishikawa T., Kakei S. (2019). Neural evidence of the cerebellum as a state predictor. Cerebellum.

[18] Tanaka H., Ishikawa T., Lee J., Kakei S. (2020). The cerebro-cerebellum as a locus of forward model: A review. Front. Syst. Neurosci..

[19] Apps R., Hawkes R., Aoki S., Bengtsson F., Brown A.M., Chen G., Ebner T.J., Isope P., Jörntell H., Lackey E.P. (2018). Cerebellar modules and their role as operational cerebellar processing units: A consensus paper [corrected]. Cerebellum.

[20] Wu H.S., Sugihara I., Shinoda Y. (1999). Projection patterns of single mossy fibers originating from the lateral reticular nucleus in the rat cerebellar cortex and nuclei. J. Comp. Neurol..

[21] Ishikawa T., Shimuta M., Häusser M. (2015). Multimodal sensory integration in single cerebellar granule cells in vivo. eLife.

[22] Voogd J. (1967). Comparative aspects of the structure and the fiber connexions of the mammalian cerebellum. Prog. Brain. Res..

[23] De Zeeuw C.I., Lisberger S.G., Raymond J.L. (2021). Diversity and dynamics in the cerebellum. Nat. Neurosci..

[24] Brandsma R., Spits A.H., Kuiper M.J., Lunsing R.J., Burger H., Kremer H.P., Sival D.A., Childhood Ataxia and Cerebellar Group (2014). Ataxia rating scales are age-dependent in healthy children. Dev. Med. Child Neurol..

[25] Berger S.E., Adolph K.E. (2007). Learning and development in infant locomotion. Prog. Brain Res..

[26] Sathyanesan A., Gallo V. (2019). Cerebellar contribution to locomotor behavior: A neurodevelopmental perspective. Neurobiol. Learn. Mem..

[27] Limperopoulos C. (2010). Extreme prematurity, cerebellar injury, and autism. Semin. Pediatr. Neurol..

[28] Reisman D.S., Wityk R., Silver K., Bastian A.J. (2007). Locomotor adaptation on a split-belt treadmill can improve walking symmetry post-stroke. Brain.

[29] Hoogkamer W., Bruijn S.M., Sunaert S., Swinnen S.P., van Calenbergh F., Duysens J. (2015). Adaptation and aftereffects of split-belt walking in cerebellar lesion patients. J. Neurophysiol..

[30] Morton S.M., Bastian A.J. (2004). Cerebellar control of balance and locomotion. Neuroscientist.

[31] Yamaguchi K., Goto N., Yamamoto T.Y. (1989). Development of human cerebellar nuclei. Morphometric study. Acta. Anat..

[32] Holland D., Chang L., Ernst T.M., Curran M., Buchthal S.D., Alicata D., Skranes J., Johansen H., Hernandez A., Yamakawa R. (2014). Structural growth trajectories and rates of change in the first 3 months of infant brain development. JAMA Neurol..

[33] Deoni S.C., Catani M. (2007). Visualization of the deep cerebellar nuclei using quantitative T1 and rho magnetic resonance imaging at 3 Tesla. Neuroimage.

[34] Zecevic N., Raki C.P. (1976). Differentiation of Purkinje cells and their relationship to other components of developing cerebellar cortex in man. J. Comp. Neurol..

[35] Hashimoto K., Ichikawa R., Kitamura K., Watanabe M., Kano M. (2009). Translocation of a “winner” climbing fiber to the Purkinje cell dendrite and subsequent elimination of “losers” from the soma in developing cerebellum. Neuron.

[36] Sugihara I. (2006). Organization and remodeling of the olivocerebellar climbing fiber projection. Cerebellum.

[37] White J.J., Sillitoe R.V. (2013). Development of the cerebellum: From gene expression patterns to circuits maps. Wiley Interdiscip. Rev. Dev. Biol..

[38] Ichise T., Kano M., Hashimoto K., Yanagihara D., Nakao K., Shigemoto R., Katsuki M., Aiba A. (2000). mGluR1 in cerebellar Purkinje cells essential for long-term depression, synapse elimination, and motor coordination. Science.

[39] Manto M.U., Jissendi P. (2012). Cerebellum: Links between development, developmental disorders and learning. Front. Neuroanat..

[40] Piochon C., Kano M., Hansel C. (2016). LTD-like molecular pathways in developmental synaptic pruning. Nat. Neurosci..

[41] Tiemeier H., Lenroot R.K., Greenstein D.K., Tran L., Pierson R., Giedd J.N. (2010). Cerebellum development during childhood and adolescence: A longitudinal morphometric MRI study. NeuroImage.

[42] Sussman D., Leung R.C., Chakravarty M.M., Lerch J.P., Taylor M.J. (2016). The developing human brain: Age-related changes in cortical, subcortical, and cerebellar anatomy. Brain Behav..

[43] Kipping J.A., Tuan T.A., Fortier M.V., Qiu A. (2017). Asynchronous development of cerebellar, cerebello-cortical, and cortico-cortical functional networks in infancy, childhood, and adulthood. Cereb. Cortex.

[44] Hartley H., Cassidy E., Bunn L., Kumar R., Pizer B., Lane S., Carter B. (2019). Exercise and physical therapy interventions for children with ataxia: A systematic review. Cerebellum.

[45] Wang S.S., Kloth A.D., Badura A. (2014). The cerebellum, sensitive periods, and autism. Neuron.

[46] Wells E.M., Walsh K.S., Khademian Z.P., Keating R.F., Packer R.J. (2008). The cerebellar mutism syndrome and its relation to cerebellar cognitive function and the cerebellar cognitive affective disorder. Dev. Disabil Res. Rev..

[47] Louis E.D. (2022). Silas Weir Mitchell on Cerebellum: Rich neurophysiological concepts and a modern perspective. Cerebellum.

